# CellRegNet: Point Annotation-Based Cell Detection in Histopathological Images via Density Map Regression

**DOI:** 10.3390/bioengineering11080814

**Published:** 2024-08-10

**Authors:** Xu Jin, Hong An, Mengxian Chi

**Affiliations:** School of Computer Science and Technology, University of Science and Technology of China, Hefei 230000, China; jinxu@mail.ustc.edu.cn (X.J.); chimengxian@mail.ustc.edu.cn (M.C.)

**Keywords:** digital pathology, cell detection, attention mechanism

## Abstract

Recent advances in deep learning have shown significant potential for accurate cell detection via density map regression using point annotations. However, existing deep learning models often struggle with multi-scale feature extraction and integration in complex histopathological images. Moreover, in multi-class cell detection scenarios, current density map regression methods typically predict each cell type independently, failing to consider the spatial distribution priors of different cell types. To address these challenges, we propose CellRegNet, a novel deep learning model for cell detection using point annotations. CellRegNet integrates a hybrid CNN/Transformer architecture with innovative feature refinement and selection mechanisms, addressing the need for effective multi-scale feature extraction and integration. Additionally, we introduce a contrastive regularization loss that models the mutual exclusiveness prior in multi-class cell detection cases. Extensive experiments on three histopathological image datasets demonstrate that CellRegNet outperforms existing state-of-the-art methods for cell detection using point annotations, with F1-scores of 86.38% on BCData (breast cancer), 85.56% on EndoNuke (endometrial tissue) and 93.90% on MBM (bone marrow cells), respectively. These results highlight CellRegNet’s potential to enhance the accuracy and reliability of cell detection in digital pathology.

## 1. Introduction

Histopathology is the gold standard for diagnosing a wide range of diseases [1]. Traditionally, pathologists analyze tissue slides under a microscope to make critical decisions. However, this diagnostic process is time-consuming and susceptible to both intra- and inter-observer variability [2]. The advent of digital pathology has enabled the creation of computer-aided diagnosis systems that are fast, accurate, and consistent [3]. These systems often leverage advanced machine learning techniques, particularly deep learning, to analyze complex histopathological images.

Deep learning-based methods have shown remarkable success across various tasks in digital pathology. Studies have demonstrated their effectiveness in tumor metastasis diagnosis [4], tumor subtyping [5], mutation prediction [5], and analyzing gigapixel pathology slides [6]. Among these applications, cell detection plays a crucial role in assisting pathologists to quantify cell populations [7] and identify abnormal cells [8]. As a fundamental step in the quantitative analysis of histopathological images [9], accurate cell detection is essential for numerous subsequent analytical processes and plays a pivotal role in enhancing the overall efficiency and accuracy of pathological diagnoses.

While deep learning methods have emerged as promising solutions for accurate cell detection [10,11,12,13], they are heavily reliant on large amounts of annotated data for training. The process of annotating cells requires the expertise of seasoned pathologists and presents significant challenges due to the small size and dense packing of cells in histopathological images. Creating detailed annotations, such as bounding boxes or segmentation masks, is particularly time-consuming and labor-intensive. Consequently, there is a pressing need for efficient and accurate cell detection methods that can alleviate this annotation burden while maintaining high performance.

To address the challenges associated with detailed cell annotations, an alternative approach that has gained attention for cell detection involves a simplified center-point annotation strategy [8,14,15]. While point annotations do not provide detailed information about cell size, shape, or precise boundaries, they offer significant advantages in the context of large-scale histopathological analysis. Firstly, it significantly reduces the time and effort required for annotation, allowing for rapid labeling of large datasets. Secondly, it provides cell location and type information, which is sufficient for tasks such as immunohistochemistry (IHC) scoring [7].

In this approach, each cell is marked by a single point at its center, rather than requiring a bounding box or segmentation mask. These point annotations are then converted into density maps, where each annotated cell location is represented by a Gaussian kernel [8,16], and the collective point annotations form the overall density map. During training, the model learns to predict this density map. At inference time, cell locations are determined by thresholding and local maxima searches on the predicted density maps.

While existing point annotation-based approaches have made commendable progress in improving annotation efficiency by significantly reducing manual effort, they remain insufficient for adapting to the unique characteristics of histopathological images. First, histopathological images exhibit a high degree of visual complexity, with cells varying in size, density, and morphological characteristics [17]. This complexity necessitates the analysis of features at different receptive fields and the integration of contextual information from multiple scales. However, current point annotation-based methods often lack effective mechanisms for capturing these intricate details and variations present in histopathological data. Second, when predicting density maps for multiple cell types, these methods typically extend the density map to multiple channels, with each channel corresponding to a specific cell type [8]. However, this approach treats the density maps of different cell types as conditionally independent, neglecting to incorporate prior knowledge regarding the spatial distribution of cells, which could provide valuable contextual information to enhance detection accuracy. Since individual cells have spatial extents and cannot occupy the same spatial location simultaneously, different cell types exhibit distinct spatial distribution patterns in histopathological images. Incorporating such spatial distribution priors can help improve the contrastiveness of predicted density maps across different cell types, reducing false positives and enhancing overall detection accuracy.

Motivated by the above discussions, we propose CellRegNet, a novel approach that leverages a hybrid CNN/Transformer architecture to effectively capture multi-scale visual cues and incorporates spatial distribution priors to guide the model’s prediction of density maps for cell detection. Specifically, CellRegNet employs a hybrid CNN/Transformer encoder to extract both local and global features, feature bridges to refine multi-scale features, and global context-guided feature selection (GCFS) blocks to select the most informative local features guided by global context. Additionally, we introduce a contrastive regularization loss that encourages the predicted density maps of different cell types to exhibit high contrastiveness, thereby reducing false positives and improving detection accuracy. Through extensive experiments on three public histopathological datasets, we demonstrate that CellRegNet outperforms existing state-of-the-art methods for cell detection using point annotations by achieving higher F1-scores. Our proposed approach effectively leverages multi-scale visual cues and spatial distribution priors, leading to more robust and accurate cell detection in challenging histopathological images.

The main contributions of this work include the following: proposing CellRegNet, a novel hybrid CNN/Transformer model for cell detection in histopathological images; incorporating feature bridges and global context-guided feature selection (GCFS) blocks to enhance multi-scale feature integration; and introducing a contrastive regularization loss to improve multi-class cell detection accuracy. These innovations effectively address the challenges of cellular complexity and spatial distribution in histopathological images, leading to significant improvements in cell detection performance.

The rest of the article is organized as follows: Section 2 provides an overview of related works in the fields of point annotation-based cell detection. Section 3 details the proposed CellRegNet approach, including the hybrid CNN/Transformer encoder, feature bridges, global context-guided feature selection (GCFS) blocks, contrastive regularization loss, and the inference procedure. Section 4 outlines the experimental setup, including the datasets used for evaluation, implementation details, and evaluation metrics. Section 5 presents and discusses the results, comparing the performance of CellRegNet against state-of-the-art methods and analyzing the effects of different components and design choices. Finally, Section 6 concludes the article by summarizing the key findings and contributions, and providing insights into potential future research directions.

## 2. Related Works

Cell detection in histopathological images is a crucial task in digital pathology, with applications ranging from cancer diagnosis to treatment planning [1,9]. This section provides an overview of the key developments in cell detection methods. These methods address various challenges inherent in cell detection, such as dealing with densely packed cells, varying cell sizes, and complex tissue structures. We first discuss the progression of regression-based cell detection techniques, followed by discussing the evolution of network architectures designed for this task.

### 2.1. Regression-Based Cell Detection

The detection of cell instances in histopathological images presents distinct challenges: cells are characteristically small in size, densely packed, and unevenly distributed across the image, while typically being annotated only with their center point coordinates to simplify the labeling process [8,17]. Under such circumstances, conventional anchor-based object detection methods face limitations [18]. Kainz et al. advocated for map-based regression approaches for detection [14], proposing to model each cell with a unimodal distribution, where pixel values represent the proximity to the nearest cell center. They argued that an ideal representation for modeling individual cells should maintain a flat background in non-cell regions while exhibiting distinct, localized peaks at cell centers. This representation enables straightforward detection through post-processing steps such as thresholding and local-maxima search.

A variety of regression targets have been explored for cell detection tasks. Sirinukunwattana et al. [19] introduced a spatially constrained CNN to regress cell probability maps, while Xie et al. [20] employed a fully residual network to regress proximity maps. Qu et al. [21] developed a regression model for Gaussian distance maps, utilizing a U-shaped architecture. To circumvent label adhesion, Liang et al. [22] introduced focal inverse distance transform maps as regression targets, along with the adoption of HRNet as a high-resolution prediction network. Li et al. [23] proposed exponential distance transform maps as regression targets. However, these approaches require sophisticated hyper-parameter tuning, which can be time-consuming and error-prone.

In contrast to these approaches, density map regression [8,15,16,18] offers several advantages. Density maps are generated using Gaussian kernels to approximate the Dirac distribution, making the values directly represent the density of cell distribution and thereby enhancing their interpretability. Additionally, Gaussian kernel requires only one hyper-parameter, σ, simplifying the tuning process. Density map regression was initially introduced by Zhang et al. [18] for the crowd counting task, which utilizes point annotations to localize individuals in public scenes and sports events. They proposed a multi-column CNN that leverages multiple convolutional columns with different receptive field sizes to capture multi-scale features for accurate crowd density estimation. Guo et al. [15] focused on density map regression for one-class cell detection using SAU-Net, which is based on U-Net with a self-attention module. Li et al. [16] highlighted the importance of high-resolution feature maps and large receptive fields for accurate density map prediction and proposed CSRNet. Huang et al. [8] extended the density map regression approach from single-class to multi-class settings and proposed U-CSRNet for cell detection. Additionally, they released the large-scale BCData dataset [8], comprising immunohistochemically stained breast cancer histopathological images, to facilitate public research in this domain. However, these approaches assume that density maps of different cell types are conditionally independent, ignoring prior knowledge of their spatial distribution.

### 2.2. Network Architectures for Density Map Regression

The design of network architectures for cell detection via density map regression has evolved significantly over the years, addressing the challenges of accurately capturing and representing the spatial distribution of cells. Key design principles include multi-scale feature extraction, large receptive fields, and high-resolution feature maps.

Multi-column convolutional neural networks [18] employ multiple convolutional branches with different receptive field sizes to capture multi-scale features. However, the reliance on a multi-branch architecture emphasizes the model’s width, limiting its capacity to extract deep features [16]. To simultaneously accommodate high-resolution feature maps and large receptive fields, CSRNet [16] integrates the first 10 layers of VGG-16 [24] with dilated convolution blocks [25]. These dilated convolution blocks allow CSRNet to expand the receptive fields and extract deeper features without sacrificing resolution. U-CSRNet [8] improves upon CSRNet by employing residual convolution blocks [26] instead of VGG blocks and introducing transposed convolutions to produce accurate and detailed density maps. CSRNet and U-CSRNet both utilize a series of stacked dilated convolution layers to capture fine details while maintaining a broader context. While effective, these approaches have their limitations. Stacking dilated convolutions may introduce gridding artifacts [27] and result in the loss of neighboring information.

Other networks designed for dense predictions can also be used for density map regression. HRNet [28], which maintains high-resolution representations throughout the network, is adopted by Liang et al. [22] to ensure that fine details are preserved during feature extraction. Inspired by HRNet, Zhang et al. [29] proposed DCLNet, which further integrates difference convolutions and deformable convolutions to localize cells. However, the use of deformable convolution introduces unsymmetrical offsets for each position in the sampling grid, which could be a drawback in applications requiring precise spatial alignment [30].

The U-shaped networks [31] have been widely adopted for cell detection and density map regression due to their effective encoder-decoder structure with skip connections. This architecture allows for the extraction of multi-scale features, combining deep semantic features with large receptive fields and high-resolution low-level details, which is beneficial for representing cellular characteristics and spatial distributions. SAU-Net [15] integrated an attention module at the encoder’s bottleneck to enhance feature representations. Li et al. [32] added gradient control and noise reduction modules to SAU-Net and proposed PGC-Net for cell detection. Qu et al. [21] combined a pre-trained ResNet [26] encoder with a U-shaped decoder to improve feature extraction capabilities. In recent years, U-shaped networks [31] have been combined with Transformers [33] to enlarge receptive fields by leveraging multi-head self-attention mechanisms [34,35,36]. These models are also applicable to density map regression tasks. While these models are effective for semantic segmentation tasks that demand precise delineation of object boundaries, in the context of density map regression, their extensive use of skip connections to propagate low-level features may introduce extraneous information, potentially hindering performance in density estimation objectives.

## 3. Method

This section presents our proposed CellRegNet for accurate cell detection in histopathological images using density map regression. The model leverages a hybrid architecture combining Swin Transformers and Convolutional Neural Networks (CNNs) to effectively capture both local and global features. We detail the model’s architecture, including the hybrid CNN/Transformer model, feature bridges to refine multi-scale features, and global context-guided feature selection (GCFS) blocks to select the most informative local features for the convolutional decoder responsible for density map generation. Additionally, we introduce the contrastive regularization loss, the ground truth generation process, and the inferencing procedure. More details are discussed next.

### 3.1. Model Architecture Overview

**Figure 1:**
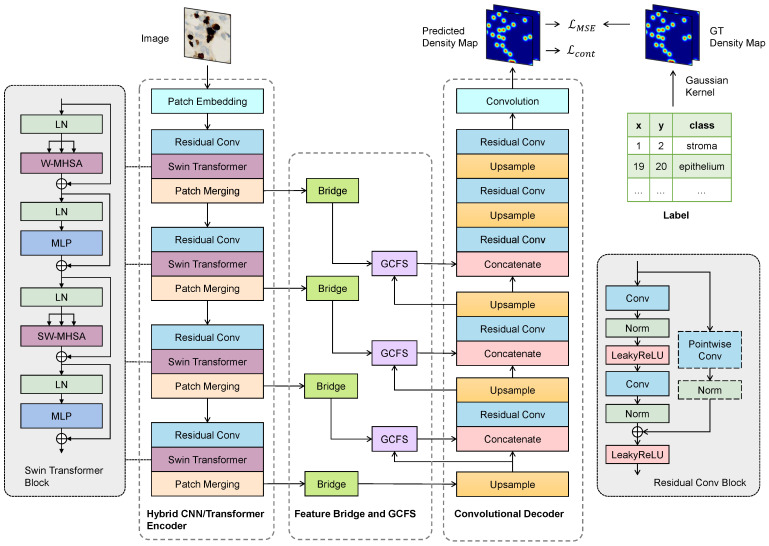
The architecture of our proposed CellRegNet for cell detection via density map regression. The model comprises a hybrid CNN/Transformer encoder for multi-scale feature extraction, feature bridges for refinement, global context-guided feature selection (GCFS) blocks for informative feature selection, and a convolutional decoder for high-resolution density map generation. Ground truth is generated from point annotations by Gaussian kernel convolutions.

The proposed CellRegNet architecture, illustrated in Figure 1, is designed to efficiently detect cells in histopathological images through density map regression. The architecture employs a U-shaped design and is composed of several interconnected modules, each contributing to the overall performance of the model.

At the core of CellRegNet is a hybrid encoder that combines Swin Transformers and convolutional neural networks (CNNs). This hybrid encoder is responsible for extracting both local and global features from the input images. The Swin Transformer captures global context and long-range dependencies, while the CNN layers focus on local feature extraction and refinement.

Following the hybrid encoder, two key components significantly enhance the model’s ability to extract and select informative features. First, feature bridges are incorporated as horizontal skip connections. These bridges enlarge the receptive field and recalibrate feature maps. Second, and notably, the global context-guided feature selection (GCFS) blocks leverage cross-attention mechanisms to select the most informative local features guided by global context.

Each of these components plays a role in the overall performance of CellRegNet and will be discussed in detail in the following sections.

### 3.2. Hybrid CNN/Transformer Encoder

This section details our hybrid CNN/Transformer encoder, which efficiently extracts multi-scale features by combining Swin Transformer modules [37] with residual convolution blocks [26].

The hybrid encoder combines convolutional neural networks (CNNs) and Transformer architectures to extract multi-scale features efficiently. This hybrid approach aims to leverage the strengths of both architectures: CNNs excel at capturing local spatial information [26,38] and encoding positional information [39], while Transformers are adept at modeling long-range dependencies [33]. In the context of cell detection in histopathological images, this combination allows for the simultaneous consideration of local cellular details and broader tissue context.

The encoder begins with a patch embedding layer, which transforms the input image into embedded non-overlapping patches. Following the patch embedding layer, the model employs four sequential Swin Transformer blocks to hierarchically extract features and progressively model long-range relations [37]. Each Swin Transformer block consists of two sub-blocks, both of which include layer normalization, window-based multi-head self-attention (W-MHSA), and a multi-layer perceptron (MLP). To produce a hierarchical representation [37], a patch merging layer is placed after each Swin Transformer block. Patch merging reduces the spatial dimensions of the feature maps from $R^{C\times H\times W}$ to $R^{2C\times \frac{H}{2}\times \frac{W}{2}}$. The number of attention heads starts from three and doubles at each stage, corresponding to the increasing embedding dimensions.

Vision Transformers excel at modeling long-range relationships, but they may converge slowly on small-scale datasets [40]. Introducing convolutional priors is a common workaround to mitigate this issue and enhance feature learning [36,41,42]. Inspired by these approaches, we prepend a residual convolution block before each Swin Transformer block. Each residual block comprises two 3×3 convolution layers, instance normalization, and ReLU activation, maintaining the feature map dimensions through identity shortcut.

The hybrid CNN/Transformer encoder effectively integrates the capabilities of convolutional and Transformer architectures. By combining convolutions with Swin Transformers, our model utilizes both local and global feature extraction mechanisms. This approach aims to enhance overall feature learning, particularly for cell detection tasks where cells can vary significantly in size, density, and morphology, and where the global tissue context provides important cues for accurate detection. The extracted features are further enhanced and recalibrated in the feature bridge, as detailed in the next section.

### 3.3. Feature Bridge

This section introduces the feature bridge, a component designed to refine and recalibrate features extracted by the encoder, with specific considerations for histopathological image analysis.

Feature bridges are lateral connections that facilitate the transfer of information from the intermediate stages of the encoder to the decoder. This mechanism promotes better gradient flow and aids the decoder in recovering details that might be lost during the downsampling process. As illustrated in Figure 1, in CellRegNet, feature bridges are employed as skip connections at the three deepest levels of the encoder, excluding the essential connection between the encoder’s final stage and the decoder’s initial stage. This design balances the preservation of informative features with computational efficiency.

In the analysis of histopathological images, accurately determining cell presence and identifying cell types requires the consideration of both fine-grained cellular details and broader tissue contexts. Motivated by recent advancements in convolutional neural networks [43,44], we incorporate large kernel depthwise convolutions into the feature bridge. This design choice aims to expand the effective receptive fields of the model [41], thereby refining the extracted features while maintaining computational efficiency. The increased receptive field allows the model to simultaneously capture local cellular characteristics and their surrounding tissue structures.

**Figure 2:**
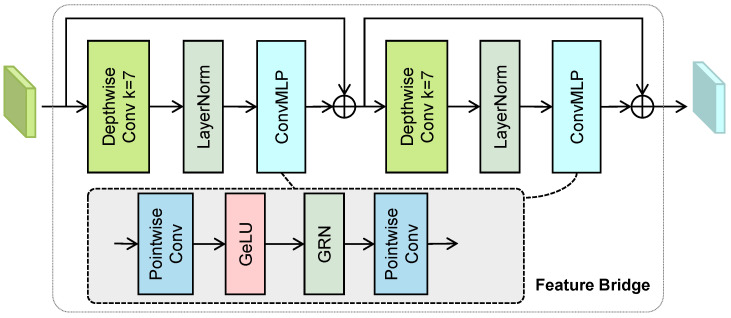
The structure of the feature bridge, which incorporates 7×7 depthwise convolutions and lightweight inverted bottleneck MLPs.

As illustrated in Figure 2, each feature bridge consists of two stacked ConvNeXt-V2 blocks [44]. Each block incorporates a depthwise 7×7 convolution, layer normalization, and a lightweight inverted bottleneck convolutional MLP implemented by pointwise convolutions and global response normalizations (GRNs). The input features first pass through a 7×7 depthwise convolution to expand the receptive field. Subsequently, a lightweight convolutional MLP, composed of two pointwise convolutions, facilitates information exchange along the channel dimension.

The GRN module, used in the convolutional MLP, recalibrates the feature maps [44]. Let $F=[f_{1},f_{2},\ldots ,f_{C}]$ be the input feature map, where *C* is the number of channels and $f_{i}\in R^{H\times W}$ denotes the feature map of the *i*-th channel. GRN first computes the L2 norm of F for each channel: $G=[g_{1},g_{2},\ldots ,g_{C}]$, where $g_{i}={∥f_{i}∥}_{2}$. The norms are then normalized across channels to compute the per-channel relative importance weights:

$$w_{i}=\frac{g_{i}}{\mathrm{mean}(g_{1},g_{2},\ldots ,g_{C})}$$

The features F are then reweighted using $W=[w_{1},w_{2},\ldots ,w_{C}]$, and adjusted using a residual connection and learnable parameters $\gamma \in R^{C}$ and $\beta \in R^{C}$:

Y=F+γ⊙F⊙W+β

where operands are broadcasted if needed and ⊙ denotes element-wise multiplication. The feature maps refined by the bridge are then passed through a global context-guided feature selection module before being integrated into the decoder. This process will be discussed in detail in the following section.

### 3.4. Global Context-Guided Feature Selection

The decoder of CellRegNet progressively integrates intermediate features from the encoder provided through feature bridges at each stage. While deep features encapsulate comprehensive global information, intermediate layer features provide essential local information to produce high-quality density map predictions. Therefore, a critical challenge is how to effectively integrate these features.

An intuitive approach is to train the model to learn to select relevant local detail features informed by the global context. Inspired by Transformers in neural machine translation tasks [33], we model this problem as a sequence-to-sequence cross-attention task, where the global-level context serves as queries, and intermediate-level features as keys and values.

**Figure 3:**
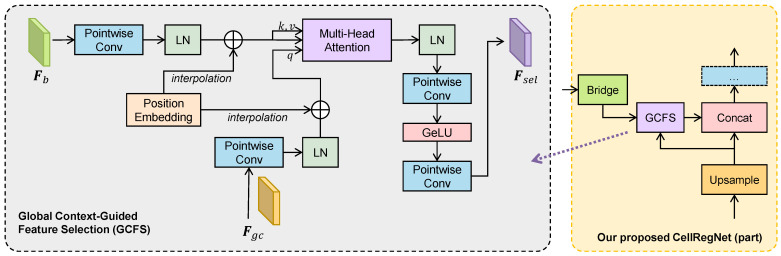
Structure and location of our proposed global context-guided feature selection (GCFS) module. Left: details of the GCFS module. Right: Part of our proposed CellRegNet, highlighting the location of GCFS. GCFS utilizes multi-head cross-attention between the global context features $F_{gc}$ and the lower-level features $F_{b}$ bridged from the encoder output, with the global context features serving as the query in the attention mechanism.

As illustrated in Figure 3, GCFS modules are situated between the feature bridges and the decoder stages. GCFS takes two input feature maps: $F_{b}$, the intermediate-level feature map from the lateral feature bridge, and $F_{gc}$, the global context information from the previous decoder stage. The input feature maps are initially processed through a pointwise convolution layer to adjust the number of channels to the embedding dimension $d_{model}$. Subsequently, the feature maps are flattened along the spatial dimension into token sequences and normalized using layer normalization. To preserve spatial relationships, learnable positional encodings are added to the flattened tokens. If the spatial size of the positional encodings does not align with the input size, bicubic interpolation is applied to adjust them accordingly.

After these preparations, the global context $F_{gc}$ is transformed into a token sequence $T_{gc}$, and the local features from the bridge $F_{b}$ are transformed into $T_{b}$. These two sequences are then fed into a multi-head cross attention module.

Specifically, for each attention head, we construct the query Q, key K, and value V through linear projection:

$$\begin{array}{cc} Q & =T_{gc}\;\cdot \;W_{q},\\ K & =T_{b}\;\cdot \;W_{k},\\ V & =T_{b}\;\cdot \;W_{v}. \end{array}$$

The attention scores are calculated by:

$$S=\mathrm{softmax}\left(\frac{{\mathrm{QK}}^{T}}{\sqrt{d}}\right),$$

where $d=d_{model}/h$ and *h* is the number of attention heads. By applying the attention scores to V according to the standard Transformer mechanism [33], we obtain the attention-reweighted local features. These reweighted local features are then normalized using layer normalization, reshaped back to their original spatial dimensions, and passed through a convolutional MLP to generate the selected local features guided by the global context.

It is worth noting that, unlike standard Transformers [33] or Vision Transformers [40], the GCFS module does not employ residual connections to bypass the multi-head attention or MLP components. This intentional design choice introduces a feature bottleneck effect, compelling the model to rigorously select and focus on essential features.

By guiding local feature selection through global context, GCFS ensures that the most relevant local features are emphasized. This approach enhances the decoder’s ability to synthesize high-quality density maps, thereby improving the model’s performance and robustness in cell detection tasks.

### 3.5. Convolutional Decoder

As illustrated in Figure 1, the decoder of CellRegNet iteratively upsamples the global-level feature map and progressively integrates the selected local-level feature maps to generate density map predictions. We employ transposed convolutional layers to upsample the feature map by a factor of 2 at each stage.

Feature integration is implemented by concatenating the feature maps along the channel dimension, followed by a residual convolution block. This approach facilitates the fusion of information across channels, smooths spatial features, and preserves fine-grained details through residual connections. The final layer of the decoder consists of a pointwise convolution with *K* output channels, where *K* represents the number of classes in the desired density map. Since we are performing linear regression in this layer, no activation function is applied.

The resulting output density map can be utilized for calculating training loss or performing inference, as will be discussed in subsequent sections.

### 3.6. Training Objective

In this section, we first introduce the details of generating ground truth density maps from sparsely annotated points. Then, we describe the loss function used for training.

#### 3.6.1. Ground Truth Generation

For each image $x\in R^{3\times H\times W}$ within the dataset D, we define its annotated points as a set A. Each element in A is a triplet (x,y,k), where *x* and *y* represent the coordinates of the cell center, and k∈{1, 2, …, K} denotes the cell type or class. Formally, if an image has a total of *N* annotated cells, we have:

$$A=\{\left(x_{i},y_{i},k_{i}\right)|i=1,\;2,\;\ldots ,\;N\}.$$

The ground truth density map for training is denoted as $y\in R^{K\times H\times W}$, which has the same spatial size as the image x and a number of channels equal to the total number of classes *K*.

Following the methods described in the literature [8,16], we use Gaussian convolution to create the ground truth y. First, we create an empty density map y initialized to zeros. Then, for each annotated point in A, characterized by its coordinates (x,y) and class *k*, we set the value at (x,y) to 1 on the *k*-th channel of y. Finally, we apply a Gaussian convolution kernel $G_{\sigma }$ to each channel of the density map. The Gaussian kernel approximates a Dirac distribution, resulting in a meaningful representation of object density that captures both the location and the spread of objects in the image.

#### 3.6.2. Loss Function

In this study, we employ the mean squared error (MSE) loss as the primary loss function for density map regression. Formally, let $y\in R^{K\times H\times W}$ denote the ground truth density maps and $\hat{y}\in R^{K\times H\times W}$ denote the predicted density maps. The MSE loss function is defined as follows:

$$L_{\mathrm{MSE}}=\frac{1}{KHW}\sum_{k=1}^{K}\sum_{i=1}^{H}\sum_{j=1}^{W}{\left({\hat{y}}_{kij}-y_{kij}\right)}^{2}.$$

While MSE is a standard loss function for density map regression tasks [8,16], it can lead to blurry density maps [22,45], which pose a significant challenge in the post-processing stage where thresholding and local maxima detection are performed to identify cell locations [14]. A blurred density map can result in higher false positive rates, as it becomes difficult to distinguish between true cell centers and noise.

Moreover, predicting density maps for different classes in separate channels and calculating MSE loss element-wise implicitly assumes that the density maps of different cell types are conditionally independent. However, in histopathological images, the density maps of different cell types exhibit inherent differences due to the fact that different types of cells cannot occupy the same spatial location.

As discussed in Section 3.6.1, cells are annotated by center points. The values on the ground truth density map are modeled by a Gaussian distribution centered at these points, which rapidly decays away from the center. We aim for the model’s output density maps to maintain similar characteristics of high central peaks and rapid decay away from the center. Therefore, if a particular location exhibits a high response in the density map of one cell type, it indicates proximity to the center of a cell of that type and is unlikely for the same location to show a significant response in the density map of another cell type. This distinction guides the design of our loss function, incorporating a prior that the density map should exhibit high contrastiveness across different channels.

Take a two-class cell density map regression task as an example. The predicted density map $\hat{y}\in R^{2\times H\times W}$ is first separated into two density maps of shape H×W. These maps are then passed through a ReLU function to ensure non-negativity, and viewed as two HW-dimensional vectors: $\vec{u}$ and $\vec{v}$, each corresponding to the density map of a class. To evaluate the contrastiveness between the two density vectors, we use their inner product:

$$\mathrm{prod}=\vec{u}\;\cdot \;\vec{v}$$

Maximizing the contrastiveness across different channels can then be achieved by minimizing this inner product. Therefore, for *K* classes where K≥2, the contrastive regularization loss is defined as:

$$L_{\mathrm{cont}}=\frac{1}{HW}\sum_{u=1}^{K}\sum_{v=u+1}^{K}\phi \left({\hat{y}}_{u}\right)\;\cdot \;\phi \left({\hat{y}}_{v}\right),$$

where ϕ(·) denotes the ReLU function, and ${\hat{y}}_{i}\in R^{HW}$ represents the flattened *i*-th channel of $\hat{y}\in R^{K\times H\times W}$.

Our proposed contrastive loss function regularizes the predicted density map by penalizing overlaps between different channels, thereby encouraging the model to produce more distinct and accurate predictions for each cell type.

The total loss function $L_{\mathrm{total}}$ is then given by:

$$L_{\mathrm{total}}=L_{\mathrm{MSE}}+\lambda L_{\mathrm{cont}}$$

where λ is a hyperparameter to control the regularization.

This combined loss function leverages the strengths of both MSE for overall density prediction accuracy and the contrastive loss for enhancing inter-class distinctions. By doing so, it addresses the limitations of using MSE alone and potentially improves the model’s ability to accurately locate and classify different cell types in histopathological images.

### 3.7. Inference

During the inference phase, the model’s output density maps undergo a series of standard post-processing steps to accurately identify cell locations. Initially, a ReLU function ensures non-negative density values. Subsequently, thresholding filters out low-intensity predictions, reducing noise and focusing on relevant regions. Finally, for each class, local maxima detection, analogous to non-maximum suppression (NMS) in object detection, pinpoints cell centers within high-intensity areas. These local maxima are considered the predicted cell centers, with their values representing confidence scores. This post-processing pipeline efficiently transforms continuous density map predictions into discrete cell location predictions, facilitating subsequent analysis and evaluation.

## 4. Experiments

In this section, we first introduce the datasets used in this study. Subsequently, we describe the evaluation metrics and the experimental setup.

### 4.1. Datasets

We evaluate the proposed CellRegNet on three publicly available datasets: the BCData dataset [8], the EndoNuke dataset [17] and the MBM Dataset [46].

#### 4.1.1. BCData Dataset

The BCData dataset [8] comprises 1338 immunohistochemical (IHC) stained breast cancer histopathological images, with manually annotated cell centers for two different cell types: Ki-67 positive and Ki-67 negative tumor cells. Ki-67 is a biomarker that is associated with cell proliferation. Accurately detecting and identifying different types of tumor cells can aid the pathologists in determining the Ki-67 expression level and guide the treatment decisions. The dataset comes with a pre-defined split into 803 training, 133 validation, and 402 test images; all images have a resolution of 640 × 640 pixels and contain various cell densities. The dataset contains a total 62,623 positive and 118,451 negative tumor cells, respectively.

#### 4.1.2. EndoNuke Dataset

The EndoNuke dataset [17] is a publicly available dataset comprising image tiles extracted from immunohistochemically (IHC) stained slides of endometrium tissue specimens. The dataset was created with the objective of facilitating the development of automated cell detection models to aid in the scoring process for endometrium IHC slides. Automating this scoring process could significantly enhance the diagnosis of infertility and other diseases [47].

The EndoNuke dataset comprises two components: the first consists of 40 image tiles used for an expert agreement study [17], while the second, which was employed in this study, contains 1740 image tiles. This latter subset includes 170,996 stromal cells and 37,722 epithelial cells, annotated as points by seven human experts. The subset was further partitioned into 1215 tiles for training, 175 for validation, and 350 for testing. Each image tile has an original physical size of 100μm×100μm, which was rescaled to 448×448 pixels to facilitate computational experiments.

#### 4.1.3. MBM Dataset

The modified bone marrow (MBM) dataset [46] is derived from the original bone marrow (BM) dataset introduced by Kainz et al. [14]. The original BM dataset comprises images of bone marrow tissues, providing a valuable resource for cell detection tasks in hematological contexts.

The MBM dataset consists of 44 non-overlapping images, each with a resolution of 600 × 600 pixels. Each image contains an average of 126 ± 33 cells. For our computational experiments, these images were rescaled to 640 × 640 pixels.

The annotations of MBM were calibrated through visual inspection with the assistance of domain experts [46], resulting in the inclusion of previously unlabeled cells. This enhancement makes it a reliable benchmark for evaluating cell detection algorithms.

### 4.2. Evaluation Metrics

To comprehensively evaluate the performance of our model, this study follows a structured process that includes matching model predictions with ground truth annotations, calculating per-class metrics, and summarizing these metrics for overall performance [8,19]. This section provides the detailed steps and methodology.

#### 4.2.1. Matching Predictions with Ground Truth

The first step in our evaluation process involves matching the model’s predictions with the corresponding ground truth annotation points. As described in Section 3.7, for each image and each class, the model’s prediction is a set of cell centers with corresponding confidence scores. We start by sorting the predicted cell centers in descending order of their confidence scores. Subsequently, we iterate through each predicted cell center and attempt to match it with the nearest ground truth (GT) point within a specified radius *r*. The details are described in Algorithm 1, following four key principles:

1. Each predicted cell center can be matched to at most one GT point.
2. Each GT point can be matched to at most one predicted cell center.
3. Predicted cell centers that do not match any GT points are considered false positives.
4. Remaining unmatched GT points are considered false negatives.

By aggregating the true positives (TP), false positives (FP), and false negatives (FN) across the entire dataset, we obtain the per-class TP, FP, and FN counters for computing the per-class metrics discussed in the next section.
**Algorithm 1** Matching predicted cell centers to ground truth points1:**Input:** $P=\{p_{1},p_{2},\ldots ,p_{n}\}$, predicted cell centers with confidence scores2:**Input:** $G=\{g_{1},g_{2},\ldots ,g_{m}\}$, ground truth points3:**Parameter:** Radius *r*4:**Output:** true positives (TP), false positives (FP), false negatives (FN)5:**Initialize:** TP ←0, FP ←0, FN ←06:Sort *P* in descending order of confidence scores7:**for** each $p_{i}\in P$ **do**8:    $nearby\_gt\leftarrow \{g_{j}\in G|\mathrm{distance}\left(p_{i},g_{j}\right)\leq r\}$ {Nearby ground truth points within radius *r*}9:    **if** |nearby_gt|=1 **then**10:      TP ← TP + 111:      Remove nearby_gt from *G* {Remove the matched ground truth point from *G*}12:   **else if** |nearby_gt|>1 **then**13:      $closest\_gt\leftarrow \arg\min_{g_{j}\in nearby\_gt}\mathrm{distance}\left(p_{i},g_{j}\right)$14:      TP ← TP + 115:      Remove closest_gt from *G* {Remove the closest matched ground truth point from *G*}16:   **else**17:      FP ← FP + 118:   **end if**19:**end for**20:FN ← size(G) {Remaining unmatched ground truth points}21:**return** TP, FP, FN

#### 4.2.2. Computing Per-Class Metrics

Based on the true positives (TP), false positives (FP), and false negatives (FN) obtained from the previous section, we compute the following per-class evaluation metrics for each cell type:

Precision measures the proportion of true positive predictions among all positive predictions made by the model. It is calculated as:

$$\mathrm{Precision}=\frac{\mathrm{TP}}{\mathrm{TP}+\mathrm{FP}}$$

Recall quantifies the proportion of actual positive instances that were correctly identified by the model. It is computed as:

$$\mathrm{Recall}=\frac{\mathrm{TP}}{\mathrm{TP}+\mathrm{FN}}$$

The F1-score provides a balanced measure that combines both precision and recall. It is defined as:

$$F1-\mathrm{score}=2\times \frac{\mathrm{Precision}\times \mathrm{Recall}}{\mathrm{Precision}+\mathrm{Recall}}$$

#### 4.2.3. Summarizing Metrics for Overall Performance

To assess the overall performance of the model across all cell types, we compute the mean precision, mean recall, and mean F1-score. These summarized metrics provide a comprehensive assessment of the model’s effectiveness in a multi-class setting. Specifically, we report the mean precision, mean recall, and mean F1-score across all cell types. These mean metrics reflect the overall performance and robustness of the model in detecting different cell types.

### 4.3. Experimental Setup

In this study, experiments were conducted under a consistent configuration. The hardware platform comprised a server equipped with two Intel Xeon E5-2695 v4 CPUs, 128 GB of RAM, and four NVIDIA Tesla V100-PCIE GPUs. The software implementation was based on the PyTorch framework (version 2.3) [48].

During training, the batch size was set to 8, and the AdamW optimizer was used with a weight decay of 0.01. The initial learning rate was 0.0005 and it decayed to 0.00001 using a cosine annealing scheduler. The number of epochs was set to 500, and early stopping was applied to prevent overfitting by monitoring the performance on the validation set. For the evaluation process described in Algorithm 1, we used a radius r of 10 pixels for all datasets, following the approach in [8].

To enhance the diversity of the dataset and speed up training, random cropping was employed. Additionally, other data augmentation techniques were applied to improve the generalization capability of the model. Table 1 summarizes the data augmentation techniques used.

## 5. Results and Discussion

In this section, we first compare our proposed CellRegNet with other state-of-the-art models on three datasets introduced in Section 4.1. Following that, we present ablation studies to evaluate the effectiveness of its components. Furthermore, we discuss the impact of the proposed loss function. Finally, we provide qualitative results to visually illustrate the model’s performance.

### 5.1. Performance Comparison

To comprehensively evaluate the effectiveness of our proposed CellRegNet, we conducted experiments on three public datasets: BCData [8], EndoNuke [17] and MBM [46]. We compared our model against nine representative models widely recognized in the field: U-Net [31], UNETR [34], SAU-Net [15], U-CSRNet [8], HRNet [28], DCLNet [29], Swin UNETR [35], Swin UNETR V2 [36], and PGC-Net [32]. This selection encompasses both convolutional neural networks (CNNs) and Transformer-based architectures, representing a spectrum of approaches from established benchmarks to recent state-of-the-art methods. These models were chosen based on their demonstrated strong performance in cell detection and related dense prediction tasks in microscopy image analysis.

The experimental results for BCData, EndoNuke and MBM datasets are presented in Table 2, Table 3 and Table 4, respectively. For BCData and EndoNuke datasets, we report the per-class and mean precision, recall, and F1-score metrics. To facilitate comparison and highlight the overall performance, the best F1-scores are emphasized in bold for each category and for the mean performance, as F1-score provides a balanced measure of precision and recall. For the MBM dataset, which contains only one class, we report the precision, recall, and F1-score for bone marrow cells. In Table 4, the number of parameters and computations of different methods are presented.

The results demonstrate that CellRegNet consistently outperforms existing state-of-the-art models across all three datasets in terms of overall F1-score. On the BCData dataset, CellRegNet achieves the highest mean F1-score of 86.38%, surpassing the next best model (Swin UNETR V2) by 0.52 percentage points (Table 2). Similarly, on the EndoNuke dataset, CellRegNet attains the highest mean F1-score of 85.56%, outperforming the next best model (DCLNet) by 0.34 percentage points (Table 3). For the MBM dataset (Table 4), CellRegNet achieves the highest F1-score of 93.90%, showing a slight but significant improvement over the next best model (U-CSRNet) which achieved 93.69%. This demonstrates CellRegNet’s effectiveness even in single-class scenarios.

As shown in Table 5, CellRegNet strikes a favorable balance between model size and computational efficiency. With 8.87 million parameters and 4.20 billion MACs, it offers a state-of-the-art F1-score while maintaining a relatively modest computational footprint compared to larger models like UNETR or HRNet.

A notable aspect of CellRegNet’s performance is its ability to achieve high precision while maintaining competitive recall, particularly in multi-class scenarios. This characteristic is attributed to the contrastive regularization mechanism employed in CellRegNet, which is designed to reduce false positives in complex, multi-class cell detection tasks. The effectiveness of this approach is evident in the results for BCData and EndoNuke datasets. We provide an detailed study of loss functions discussed in Section 5.3.

The MBM dataset presents an interesting contrast as it is a single-class scenario. As shown in Table 4, while CellRegNet does not achieve the highest precision (93.19% compared to U-CSRNet’s 93.24%), it maintains a better balance between precision and recall, resulting in the highest overall F1-score of 93.90%.

The consistent balanced performance of CellRegNet across different cell types, datasets, and detection scenarios (both multi-class and single-class) demonstrates its versatility and robustness. This can be attributed to its carefully designed architectural components, including the combination of CNNs and Transformers to capture both local and global features crucial for accurate cell detection. The addition of contrastive regularization further enhances the model’s ability to distinguish between different cell types in multi-class scenarios, resulting in fewer false positive identifications.

### 5.2. Ablation Study

To analyze the contributions of different components in CellRegNet, we conducted ablation studies on the BCData dataset. We evaluated three configurations:

- Base Model: Includes the hybrid CNN/Transformer encoder and convolutional decoder described in Section 3.2 and Section 3.5, using identity shortcuts as horizontal skip connections at the three deepest levels of the encoder.
- Feature Bridge Model: Enhances the base model with feature bridges as horizontal skip connections, as described in Section 3.3.
- CellRegNet Model: Incorporates the global context-guided feature selection (GCFS) module described in Section 3.4 to select the most pertinent local features based on global information.

The performance of each configuration was evaluated using precision, recall, and F1-scores. Detailed results are presented in Table 6.

The ablation results demonstrate the effectiveness of each component in CellRegNet. The base model, which utilizes the hybrid CNN/Transformer encoder and convolutional decoder, achieves a solid mean F1-score of 85.79%. This performance underscores the strength of combining Swin Transformer [37] with convolutional priors [36,41,42] for cell detection tasks.

Adding the feature bridges significantly improves the model’s performance, increasing the mean F1-score to 86.00%. This improvement can be attributed to the enhanced information flow between the encoder and decoder, facilitated by the feature bridges. The large kernel depthwise convolutions and lightweight inverted bottleneck MLPs in the feature bridges effectively refine the extracted features by enlarging the effective receptive fields. This is particularly beneficial for histopathological image analysis, where capturing both local details and broader contextual information is crucial.

The full CellRegNet model, incorporating the GCFS module, achieves the best performance with a mean F1-score of 86.38%. The GCFS module’s ability to select the most relevant local features guided by global context proves to be highly effective in improving cell detection accuracy. Notably, the GCFS module contributes to balanced improvements on both positive and negative cell classes. For positive cells, it increases the F1-score from 86.43% to 86.74%, while for negative cells, the improvement is from 85.57% to 86.03%. This performance gain demonstrates the GCFS module’s effectiveness in selecting pertinent features for different cell types, addressing the challenge of integrating global and local information in histopathological image analysis.

### 5.3. Comparison of Loss Functions

To assess the efficacy of our proposed contrastive regularization in conjunction with the commonly used mean squared error (MSE) loss, we conducted experiments using CellRegNet on both BCData and EndoNuke datasets. Table 7 and Table 8 present the detailed results.

The results demonstrate that incorporating contrastive regularization ($L_{cont}$) alongside MSE loss ($L_{MSE}$) yields consistent improvements on both datasets. On the BCData dataset (Table 7), the addition of $L_{cont}$ enhances the mean F1-score from 86.14% to 86.38%, with notable improvements in precision for both positive and negative tumor cells. Similarly, for the EndoNuke dataset (Table 8), the combined loss function improves the mean F1-score from 85.29% to 85.56%, with substantial gains in precision for both stroma and epithelium classes.

These findings align with the intended purpose of the contrastive regularization term. By incorporating the domain prior of spatial distribution of cells and penalizing overlaps between different channels in the predicted density maps, $L_{cont}$ encourages the model to produce more distinct and sharper density maps for different cell types. This is reflected in the consistent improvement in precision and F1-score across all cell types and datasets, indicating that the model becomes more discriminative in identifying true cell locations.

### 5.4. Qualitative Results

**Figure 4:**
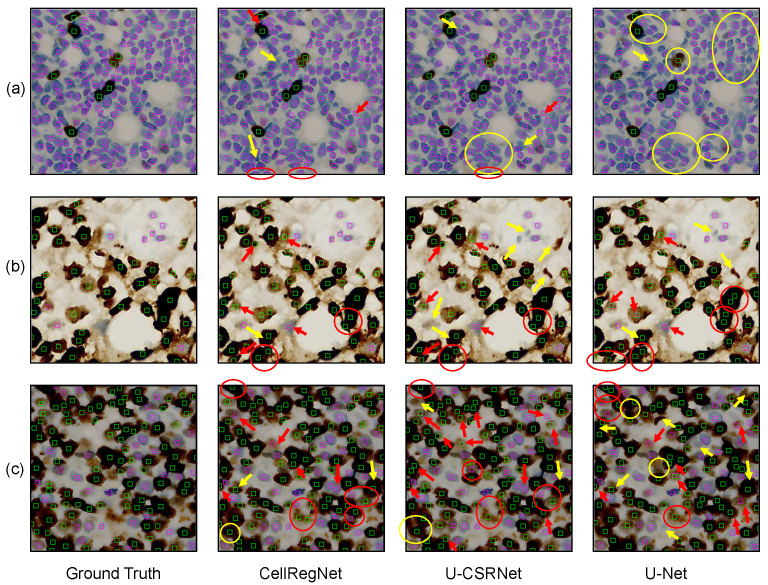
Qualitative comparison of cell detection results. Rows (**a**–**c**) visualize three representative cases. For each case, ground truth, CellRegNet predictions, U-CSRNet predictions, and U-Net predictions are illustrated. Green boxes indicate positive tumor cells, magenta boxes indicate negative tumor cells. Red markers indicate false positives and yellow markers indicate false negatives.

To visually illustrate the performance of CellRegNet, we present exemplary cell detection results on the BCData dataset. Figure 4 showcases a comparative analysis across three representative cases. Each row represents a case, with the columns displaying the ground truth (GT), CellRegNet detection results, U-CSRNet results, and U-Net results, respectively. U-CSRNet [8] and U-Net [31] serve as benchmarks for comparison. To facilitate comprehension, we have highlighted representative mispredictions in the images: red markers indicate false positives, and yellow markers indicate false negatives. It is worth noting that when a cell of class A is misclassified as class B, it is counted as both a false negative for class A and a false positive for class B in our quantitative evaluation metrics.

As evidenced by Figure 4, CellRegNet demonstrates a robust capacity for accurate and comprehensive cell detection in Ki67-stained breast cancer images. The comparative analysis reveals several observations across the different models. U-Net shows relatively lower performance in scenarios with densely packed cells, as highlighted by the yellow circle in the upper right corner of Figure 4a. This indicates the challenges faced by simpler architectures in complex, crowded cellular environments. U-CSRNet demonstrates improved ability compared to U-Net. However, it shows increased false negatives in certain scenarios, particularly in areas with blurred color due to staining or scanning artifacts, and cells with irregular morphologies (yellow arrows in Figure 4b).

The correct detection and classification of individual cells in histological images remains a challenging problem, especially in scenarios where cells are densely packed or where multi-layered cellular structures are projected onto a 2D plane. This challenge is evident in Figure 4b, where all three models exhibit some false positives in areas where cells appear to be overlapping or in close proximity. These overlapping appearances could be due to actual cell adhesion in the tissue or the result of multiple cell layers being captured in the 2D projection of the histological section, leading to visual ambiguities in the image. In scenarios with crowded and dense cell populations (Figure 4c), correct identification and classification of individual cells pose significant challenges. While CellRegNet shows relatively better performance in these complex scenarios, there is still room for improvement in handling difficult samples caused by cell crowding, tissue architecture complexity, and staining variability.

### 5.5. Discussion

The experimental results demonstrate that CellRegNet consistently outperforms existing state-of-the-art models in cell detection tasks across three distinct histopathological datasets. On the BCData dataset, CellRegNet achieves a mean F1-score of 86.38%, surpassing the next best model by 0.52 percentage points. Similarly, on the EndoNuke dataset, it attains a mean F1-score of 85.56%, outperforming the closest competitor by 0.34 percentage points. For the MBM dataset, CellRegNet achieves the highest F1-score of 93.90%, showing an improvement over the next best model by 0.21 percentage points. These improvements are consistent across different cell types, indicating the model’s robustness and versatility in handling diverse histopathological images.

The superior performance of CellRegNet can be attributed to several key factors. First, the hybrid CNN/Transformer architecture enables effective capture of both local details and global context, which is crucial for accurate cell detection in complex histopathological images. The combination of convolutional layers and Swin Transformers addresses the need for multi-scale feature extraction—a challenge often encountered in previous methods. Second, the feature bridges contributes to more accurate density map predictions by significantly enlarge the receptive field and calibrate multi-scale features, enhancing information flow between the encoder and decoder. Third, the global context-guided feature selection (GCFS) module effectively selects the most informative local features guided by global context, addressing the challenge of integrating multi-scale information in histopathological image analysis.

Furthermore, the contrastive regularization loss introduces a valuable mechanism for modeling the mutual exclusiveness prior of different cell types within the learning process. By encouraging distinctiveness between density maps of different cell types, this loss function enhances the model’s ability to capture the spatial relationships and relative occurrences of different cell types. This approach improves the model’s discriminative power and contributes to a more accurate representation of the cellular landscape in histopathological images.

The results indicate that CellRegNet demonstrates improved accuracy in cell detection for histopathological image analysis. This approach has the potential to assist pathologists in their diagnostic work by enhancing biomarker quantification and automating cell counting tasks, which may contribute to more consistent results and reduce manual workload. Future research may focus on optimizing the model architecture to balance performance and computational efficiency, as well as validating its capabilities and limitations across various clinical contexts.

## 6. Conclusions

In this study, we introduced CellRegNet, a novel deep learning model for cell detection in histopathological images using point annotations. The key contributions of this work are as follows:

1. We propose CellRegNet, a novel hybrid CNN/Transformer model for accurate cell detection in histopathological images using point annotations. CellRegNet effectively captures and integrates multi-scale visual cues, addressing the complexity of cellular structures in histopathological tissues.
2. We introduce feature bridges as horizontal skip connections, which enlarge the receptive field and recalibrate feature maps. This innovation enhances the model’s ability to capture and leverage information across various scales.
3. We design global context-guided feature selection (GCFS) blocks that leverage cross-attention mechanisms. These blocks enable the model to select the most informative local features guided by global context, significantly improving cell detection accuracy.
4. We propose a contrastive regularization loss that incorporates spatial distribution priors of cells, enhancing the distinction between predicted density maps of different cell types and reducing false positives in multi-class cell detection.

CellRegNet demonstrates state-of-the-art performance on three public histopathological datasets, showcasing its effectiveness in accurately detecting and classifying cells in challenging scenarios. The model’s robust performance underscores its potential for real-world applications in digital pathology, potentially aiding pathologists in more efficient and accurate diagnoses.

Future research directions may include optimizing computational efficiency to facilitate real-time analysis, exploring the model’s adaptability to a broader range of histopathological image types, and investigating the integration of CellRegNet into comprehensive digital pathology workflows.

## Figures and Tables

**Table 1:** Data augmentation techniques used in the experiments.

Method	Parameters
Random Crop	Crop size = 256
Random Flip	Randomly chosen from {not applied, horizontal, vertical}
Random Rotation	Randomly chosen from {0°, 90°, 180°, 270°}
Random Color Jitter	Probability = 0.5, Brightness = 0.2,
	Contrast = 0.2, Saturation = 0.1, Hue = 0.05

**Table 2:** Comparing our proposed method with representative studies on the BCData dataset. All values are in percentage, higher is better, and the best F1-scores are highlighted in bold.

Method	Positive			Negative			Mean		
	P (%)	R (%)	F1 (%)	P (%)	R (%)	F1 (%)	P (%)	R (%)	F1 (%)
U-Net [31]	84.49	86.43	85.44	84.31	82.88	83.59	84.40	84.65	84.52
UNETR [34]	84.49	85.18	84.83	81.94	82.39	82.16	83.21	83.78	83.50
SAU-Net [15]	84.15	88.03	86.05	82.83	85.66	84.22	83.49	86.84	85.13
U-CSRNet [8]	84.68	87.65	86.14	85.89	84.05	84.96	85.29	85.85	85.55
HRNet [28]	83.95	87.99	85.93	85.08	84.14	84.60	84.51	86.07	85.27
DCLNet [29]	84.24	88.24	86.19	84.10	85.25	84.67	84.17	86.74	85.43
Swin UNETR [35]	83.93	87.90	85.87	83.30	86.08	84.67	83.62	86.99	85.27
Swin UNETR V2 [36]	85.09	87.94	86.50	84.83	85.63	85.23	84.96	86.79	85.86
PGC-Net [32]	85.15	87.51	86.31	82.59	87.16	84.81	83.87	87.33	85.56
Proposed CellRegNet	86.37	87.12	**86.74**	84.11	88.03	**86.03**	85.24	87.58	**86.38**

**Table 3:** Comparing our proposed method with representative studies on the EndoNuke dataset. All values are in percentage, higher is better, and the best F1-scores are highlighted in bold.

Method	Stroma			Epithelium			Mean		
	P (%)	R (%)	F1 (%)	P (%)	R (%)	F1 (%)	P (%)	R (%)	F1 (%)
U-Net [31]	83.25	90.58	86.76	72.09	79.25	75.50	77.67	84.92	81.13
UNETR [34]	80.92	88.49	84.53	68.49	68.67	68.58	74.70	78.58	76.56
SAU-Net [15]	84.51	90.34	87.33	75.49	79.85	77.61	80.00	85.09	82.47
U-CSRNet [8]	85.78	90.46	88.06	82.43	79.04	80.70	84.10	84.75	84.38
HRNet [28]	86.00	91.12	88.49	80.08	82.94	81.48	83.04	87.03	84.99
DCLNet [29]	84.22	92.13	88.00	81.12	83.82	82.45	82.67	87.97	85.22
Swin UNETR [35]	84.88	90.97	87.82	79.24	81.33	80.27	82.06	86.15	84.05
Swin UNETR V2 [36]	85.50	91.30	88.30	80.12	83.19	81.63	82.81	87.24	84.96
PGC-Net [32]	84.80	90.80	87.70	79.85	80.04	79.94	82.32	85.42	83.82
Proposed CellRegNet	86.51	90.73	**88.57**	84.28	80.88	**82.54**	85.39	85.81	**85.56**

**Table 4:** Comparing our proposed method with representative studies on the MBM dataset. All values are in percentage, higher is better, and the best F1-score is highlighted in bold.

Method	Bone Marrow Cells		
	Precision	Recall	F1
U-Net	87.18	95.39	91.10
UNETR	88.96	95.20	91.97
SAU-Net	92.72	94.24	93.47
U-CSRNet	93.24	94.14	93.69
HRNet	90.42	96.16	93.20
DCLNet	89.77	96.06	92.81
Swin UNETR	90.87	95.58	93.16
Swin UNETR V2	91.23	95.97	93.54
PGC-Net	92.65	94.43	93.53
Proposed CellRegNet	93.19	94.62	**93.90**

**Table 5:** Comparison of number of parameters and multiply-accumulate computations (MACs) across models. The MACs are measured with an 256×256 sized RGB image and batch size of 1.

Method	# Params (M)	# MACs (G)
U-Net	0.66	0.71
UNETR	87.71	4.71
SAU-Net	2.26	10.72
U-CSRNet	10.13	11.42
HRNet	72.49	95.56
DCLNet	68.70	35.70
Swin UNETR	1.59	0.95
Swin UNETR V2	7.18	4.41
PGC-Net	2.27	11.32
CellRegNet	8.87	4.20

**Table 6:** Ablation study of our proposed CellRegNet. The best F1-scores are highlighted in bold.

Component			Positive			Negative			Mean		
Base	Bridge	GCFS	P (%)	R (%)	F1 (%)	P (%)	R (%)	F1 (%)	P (%)	R (%)	F1 (%)
✓			86.97	85.37	86.16	83.86	87.04	85.42	85.41	86.21	85.79
✓	✓		85.55	87.32	86.43	84.24	86.93	85.57	84.90	87.12	86.00
✓	✓	✓	86.37	87.12	**86.74**	84.11	88.03	**86.03**	85.24	87.58	**86.38**

**Table 7:** Evaluation of the proposed contrastive regularization loss on BCData dataset. The best F1-scores are highlighted in bold.

Loss Function		Positive			Negative			Mean		
$L_{\mathrm{MSE}}$	$L_{\mathrm{cont}}$	P (%)	R (%)	F1 (%)	P (%)	R (%)	F1 (%)	P (%)	R (%)	F1 (%)
✓		85.64	87.66	86.64	82.14	89.44	85.63	83.89	88.55	86.14
✓	✓	86.37	87.12	**86.74**	84.11	88.03	**86.03**	85.24	87.58	**86.38**

**Table 8:** Evaluation of the proposed contrastive regularization loss on EndoNuke dataset. The best F1-scores are highlighted in bold.

Loss Function		Positive			Negative			Mean		
$L_{\mathrm{MSE}}$	$L_{\mathrm{cont}}$	P (%)	R (%)	F1 (%)	P (%)	R (%)	F1 (%)	P (%)	R (%)	F1 (%)
✓		85.76	91.24	88.41	81.55	82.79	82.16	83.65	87.01	85.29
✓	✓	86.51	90.73	**88.57**	84.28	80.88	**82.54**	85.39	85.81	**85.56**

## Data Availability

Data are contained within the article.
